# Supplementary material for: Antibiofilm agents with therapeutic potential against enteroaggregative Escherichia coli
Source: PLoS Negl Trop Dis. 2022 Oct 6;16(10):e0010809. doi: 10.1371/journal.pntd.0010809 (PMC9578610; doi:10.1371/journal.pntd.0010809)
Supplement: S1 Fig — Activity was highest and concentration dependent at earlier time points; 4 and 8 h confirming that hits more likely inhibit target(s) critical to early-stage biofilm formation in EAEC. (PPTX) [file pntd.0010809.s004.pptx]

## Slide 1
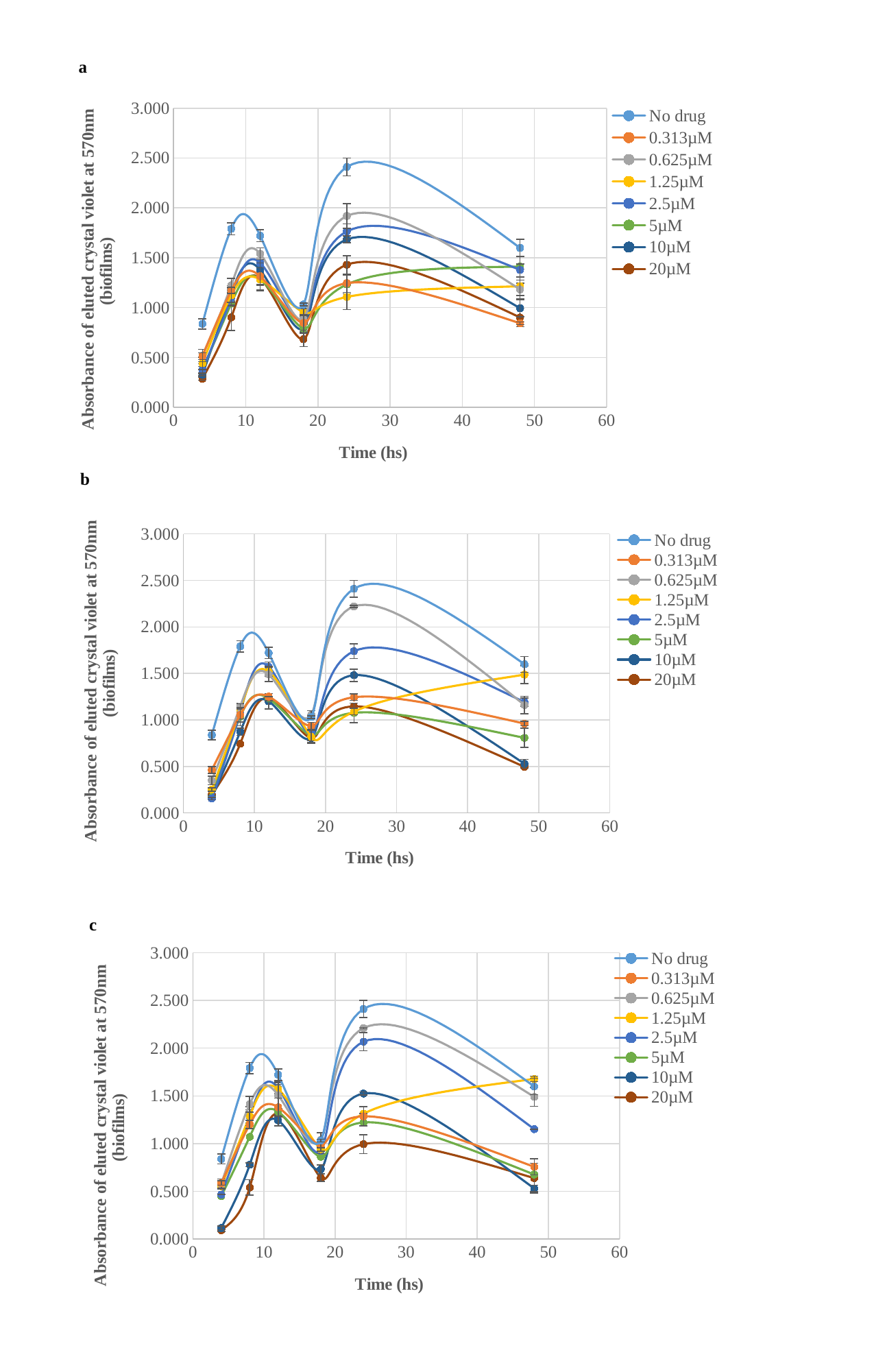

### Chart
| Category | No drug | 0.313µM | 0.625µM | 1.25µM | 2.5µM | 5µM | 10µM | 20µM |
|---|---|---|---|---|---|---|---|---|
### Chart
| Category | No drug | 0.313µM | 0.625µM | 1.25µM | 2.5µM | 5µM | 10µM | 20µM |
|---|---|---|---|---|---|---|---|---|
### Chart
| Category | No drug | 0.313µM | 0.625µM | 1.25µM | 2.5µM | 5µM | 10µM | 20µM |
|---|---|---|---|---|---|---|---|---|
