# Supplementary material for: Antibiofilm agents with therapeutic potential against enteroaggregative Escherichia coli
Source: PLoS Negl Trop Dis. 2022 Oct 6;16(10):e0010809. doi: 10.1371/journal.pntd.0010809 (PMC9578610; doi:10.1371/journal.pntd.0010809)
Supplement: Attachment — Submitted filename: PB screen DAK reviewers responsev7.docx [file pntd.0010809.s005.docx]

**Response to Reviewers comments**

| **Reviewer’s comments** | **Authors’ responses** |
| --- | --- |
| **REVIEWER 1**  **Key Review Criteria Required for Acceptance**  For the initial screen, it makes sense to add each compound concurrently with the bacteria for biofilm and growth assays. However, in the additional validation experiments for the most promising compounds, it would be stronger to add each drug to an established biofilm of EAEC (to model administration of the treatment post-infection). The authors could take the validation a step further by including intestinal cells, intestinal flora, etc. to test if these compounds remain effective in a model more similar to the gut environment. Did the formation of biofilms on the surfaces of the well interfere with the OD595nm read to measure bacterial growth? If so, was there a way to adjust for this?   1. Did the formation of biofilms on the surfaces of the well interfere with the OD595nm read to measure bacterial growth? If so, was there a way to adjust for this?   **Results**   1. Fig.5 is a key figure for the study, but the statistical comparisons for these experiments and their p-values are not all shared and their representation on the figures is confusing. The text and figure legends are unclear as to if the significant comparisons are between the treated and untreated condition for each strain, or if the measured reduction with treatment was compared between strains or to the WT condition. 2. Error bars appear quite small suggesting precise data, but it is not specified whether they are SEMs, SD, etc. Additionally, two graphs are labeled as (c) - rather than one as (b) and one as (c). These issues made it difficult to evaluate the data presented. 3. Incomplete presentation of the statistical analyses and disclosure of what the error bars represent needs to be addressed throughout but most importantly in Fig.s 5 and 6.   **Conclusions**  The conclusions are generally well supported and discussed well.   1. Inclusion of multiple, diverse EAEC isolates (Table 3) strengthens the impact of this study. The analysis of which strains responded to which compounds and the genes and alleles in those strains supports the hypothesis of MMV687800’s interaction with aaf and aap. These findings suggest that a combination treatment of different biofilm inhibitors could have broad spectrum effects on diverse EAEC strains. Was a mixed treatment considered or tested?  2.The authors speculate that because their choice compounds are not bactericidal, selective pressure for resistance will be reduced. However, within a culture, if some bacteria are successful in maintaining a biofilm and thus are better able to replicate, that could be sufficient to select for resistors. In Fig.4a, we see a decrease in biofilm inhibition across the first 18 hours for three different compounds. To verify the idea that resistors are not arising, bacteria can be sampled across the time lapse of the infection and sequenced for SNPs or tested for resistance ability.  3.The authors speculate that MMV687800 will be specific for EAEC due to its proposed specificity to *aap* and *aafA*. This could easily be tested by measuring the effect of MMV687800 on the growth of a nonpathogenic E. coli strain and on biofilm formation of a different type of bacteria that creates biofilms (like UPEC).  Some of the hits in this study which do not appear to interact with aap or aafa may also have effects on other biofilm-forming E. coli.  **Editorial and Data Presentation Modifications**   1. A color-coded system could improve Table 3 by using color to depict positive vs. negative values, color coding all values greater than the cut-off of 30% biofilm inhibition, or a gradated color system that corresponds to the size of the value. 2. There is a typo in the figure legend of Fig 4. Should be than instead of then | 1. The reviewer’s suggestion that we test the ability of each drug to dismantle a pre-formed biofilm is a good one and models, as the reviewer describes, the potential for the hits to be used as treatments for EAEC infection.   Page 9-10, lines 226-233, we have investigated hit activity against established (pre-formed) EAEC biofilms and results have been presented in pages 16, lines 414-420 and page 41.  We do not have the capacity to culture intestinal cells in our laboratory but agree that this could be a valuable future experiment and one that should be performed on lead compounds developed from these hits. The compounds were not active against commensal controls in the screen (line 339) and we agree that a broader spectral study including commensal and other pathogens (including more EAEC) needs to be performed once mechanisms of action of all the hits are known. This is in our experimental plan for the future.   2. Biofilm formation on the surface of wells would normally interfere with OD595nm read to determine bacterial growth. For this reason, planktonic growth was only measured after pipetting a 100µl aliquot of the 200µl culture from the wells without touching the sides into a fresh plate before measuring optical density at OD595nm. This way, interference by biofilm cells in OD determination were by-passed. This is more clearly spelled out on line 182-183 in response to the reviewer’s query. 1. Page 38, Significant comparison in Fig 5 is between treated and untreated control for each strain, the statistical tool used is the student-T test, and this was to investigate the difference between biofilm formation in treated wells and untreated controls for each strain (mutants as well as the wild type). After statistical analysis, using a level of significance set at 0.05, outcomes were used to preliminarily determine genes that are likely targets for each hit. Legend is now adjusted to define statistical comparison with the sentence “Statistical comparison was achieved between treated and untreated controls for each strain using student T-test, and errors bars are standard deviations between replicates for each determination”. 2. In response to the reviewer’s suggestion, the graphs in Figure 5, page 38 have been re-labelled as b and c. 3. Error bars on all figures represent the standard deviation among replicates and in response to the reviewer’s comment, this is explicitly spelt out where appropriate. (Page 38 and 39, legends in Figure 5 and 6 and all through Figures (in pages 34, 35, 37, and 41).   We thank the reviewer for this positive feedback   1. We agree that testing our combinations of biofilm inhibitors, as the reviewer suggests, or even biofilm inhibitors and antibacterials, could yield some interesting and valuable results. However, the mode of action of majority of the hits remains to be determined and would greatly enhance interpretation of the results of combinatorics. For this reason, this suggestion was not implemented in the revision but could be a direction for future research. In response to the reviewer’s comment, we have also added a point about this in the discussion (line 480-482 and 603-605– same as refers to Reviewer 2 point 7). 2. While it is theoretically less likely than for antibacterials, we agree with the reviewer that it is possible that mutants with resistant biofilms could evolve. In response to the reviewer’s comment, we have tempered our speculation in the discussion (page 20, line 510) Screening for mutants would help to measure resistance evolution rates. However, this is somewhat resource intensive (e.g. requiring us to sequence at least several dozen to several hundred isolates from a starter culture) and easier to test experimentally when mechanisms of action are better known. 3. We included a non-pathogenic *E. coli* strain in our initial screen and saw no activity, which is why we began to explore an EAEC-specific mechanism. To resasure the revieer, antibiofilm activities of MMV687800 have additionally been investigated against seven non-pathogenic EAEC strains isolated from healthy controls in our laboratory in Nigeria. The results showed virtually no inhibition or less than 30% inhibition in all cases for MMV687800 but some inhibition for two of the other hits. Results are shown below for review and in response to the reviewer’s request are attached to the manuscript as supplementary data and mentioned on lines 413-415 Eight non-pathogenic strains do not cover the full diversity in the species and as the reviewer states, it would be valuable to screen other pathotypes as well. As we do not have sufficient compound to do this, we did not incorporate this finding into the paper and instead wrote in line 600-602 the importance of conducting full spectral screens   Percentage biofilm inhibition by hits on non-pathogenic EAEC strains     1. Page 45, Table 3 has now been color-coded to indicate biofilm inhibition cut-off used for the analysis. 2. Page 37, then in the legend for Figure 4 has been changed to than. |
|  |  |
| **REVIEWER 2** |  |
| **Editorial and Data Presentation Modifications**   1. Minor editorial suggestions: p. 9 second paragraph second line: "eluted" not "eluded". 2. p. 12 Methods - Statistical analysis: Suggest clarify and expand to provide additional detail. 3. pp. 25-30 References. Suggest using a reference manager to better standardize the references. Several of the references have errors in spelling of author names.   p. 35. Figure 5. Suggest checking placement on graphs of the * indicating p<0.05. | 1. Page 9, line 210, eluded is now changed to eluted. 2. Page 12, line 304 to 305, statistical analysis now expanded to provide additional details. The new sentence now reads “Data from biofilm inhibition assays (average of three replicates) were analyzed by comparing inhibition / percentage inhibitions for treated and untreated controls and significant differences were inferred from Fisher’s exact test (to identify genes which are unique to strains whose biofilms were inhibited by hits) and student’s t-test analysis (for biofilm inhibition assay). Error bars were derived from standard deviations between replicates for each determination.”. 3. Page 27-33, all references have been retrieved from PubMed, verified, and corrected where necessary.   Page 38, Figure 5, * now moved from LV1 to SB1. |
|  |  |
| **REVIEWER 3** |  |
| **Summary and General Comments**   1. Regrettably, the authors did not include line numbers in their manuscript, so I will attempt to describe the position using page numbers only. 2. It has been well established over many years that the use of so-called “dynamite” or “plunger” bar plots to describe continuous distribution data is unhelpful and in many cases misleading, since they hide the true distribution of the individual samples (e.g. see Drummond & Vowler, 2011, Br J. Pharmacol https://www.ncbi.nlm.nih.gov/pmc/articles/PMC3087125/). Moreover, error bars can also be very misleading, and in the case of the various figures in this manuscript which use them (Figures 1, 4, 5, 6, 7), the authors do not even state what those error bars represent (are they standard deviation, standard error, 95% confidence intervals, or something else?). The authors must replace all bar/dynamite/plunger plots in their figures with a more appropriate figure type that adequately shows the distribution of sample values, e.g. boxplot, dotplot, or beehive plot. 3. I find some elements of the workflow for identifying and validating compounds difficult to follow at times. For example, on page 13, the authors write that “the three unvalidated hits… …in this study are kinetoplastid inhibitors”. As I read this, my thought was “what 3 hits?” – this is the first mention of these 3 hits in the text. I understand that this is described in the Figure 1 workflow, as well as later in the manuscript, but it is an abrupt introduction suggesting the text has been rearranged at some-point. This section would benefit from rewording to make the workflow much clearer for the reader, rather than relying on figures or introducing points out of order. 4. Page 14 – why did the authors choose to test the concentration dependency of only 3 out of 4 active compounds against strain 042? Compound MMV687697 is missing from this analysis, but there is no explanation for this, relating to my earlier point about the workflow being unclear in places. 5. Page 14 – there is a single sentence paragraph that describes how the planktonic cell growth of test strains was not significantly different from controls. However, is it not true that this is because the authors specifically screened and excluded any compounds that resulted in >10% growth reduction? The sentence appears to highlight a result that was inevitable. If the purpose of this sentence is to confirm a previous result, perhaps this should be moved elsewhere? 6. Page 14 – the authors state (with reference to Figure 3) that “hits exhibited potent activity, with concentrations as low as 2.5µM inhibiting biofilm formation by up to 30%”. However, examination of Figure 3 suggests that this was only for MMV687800, and that the other drugs may actually have inhibited biofilm formation more than this. Since this is an important result, it would be clearer if the authors expanded this section to describe the individual effects for each drug. 7. Figure 1 and elsewhere – it appears that some of the compounds screened resulted in a negative growth inhibition, which presumably means they enhanced the bacterial growth rates. This includes two of the authors’ five “hit” compounds, albeit the value is small for these. Is it a concern that a biofilm inhibitor proposed as a potential future therapeutic should enhance bacterial growth rates? This should be addressed in the manuscript, ideally with additional analysis/testing, but at a minimum with some discussion around this issue. 8. Table 1 provides details for the five selected compounds, but this table also provides percentage biofilm inhibition and growth inhibition. To which strain do these values pertain? There is no range given for replicates, or for the different strains tested. Is this a mean value for both strains, or a mean value for one of the strains, or something else? This table needs more detail and better labelling.   Minor Comments: 1. I would like some clarification on the actual amount of DMSO used in these experiments. On page 7, the authors state that final concentration of DMSO did not exceed 1%. However, it is not entirely clear if this is correct, and this will depend on the v/v concentration in the stock solution. For most of the biofilm inhibition assays conducted (described on page 7), it appears that the authors added 5µl drug dissolved in DMSO to 195µl of culture media. This would be 2.5% v/v, assuming DMSO represented a substantial portion of the drug stock solution. Moreover, the authors also performed 2-fold serial dilution of their drug stocs (see end of page 8) – was this performed in DMSO or DMEM, since if in DMSO this would ensure that DMSO represented the largest portion of that 2.5%? Given that their control wells were conducted using 1% v/v DMSO (2µl DMSO + 198µl media), this is a potential concern. 2. The authors describe making use of pre-existing whole genome sequences for the 25 samples from their lab they used for validation of their compounds, inferring allelic states in various virulence genes. However, there is no description of the genome sequencing or analysis, and the reference cited (Akinlabi 2019) appears to point to a conference abstract – there is no way to understand what sequencing or bioinformatic methods were used, and there are no accession numbers provided for these genomes. Please include more detail, including SRA/ENA accession numbers. 3. Minor typo, end of page 7 (line numbers would have been helpful here) – “drug solutions dissolved in net DMSO” – I presume the authors mean “neat” DMSO?   Minor typo, page 19 (end) – “Low et al (2017) in screen to identify TB compounds…” might need the addition of an ‘a’ to that phrase. | 1. The resubmitted version has been modified to include line numbers. We apologise for reviewer inconvience caused by not including them in the original version. 2. Page 42, Figure 9, plot has been converted to a box plot and the Figure 8 includes the raw data used to compute medians.Page 40 Figures 7 (a-b), additional experiment now included to clearly indicate that MMV687800 majorly targets *aap* and has *aaf* as a minor target. Error bars have also been defined clearly to state what they represent. 3. Paragraph in page 13, now moved to page 14, lines 345 to 348 where it fits best and gives clearer explanations. For clarity purposes, the compound identity codes have also been indicated in parenthesis. 4. Follow up assays as well as concentration dependent assays were carried out for the hits for which we had sufficient amount of compounds. Unlike the other three 042 hits, MMV687696 is particularly a novel compound from MMV, and we could not procure it commercially. A sentence explaining this is found in page 22, lines 564-565. 5. As indicated on page 14 lines 399 to 341, we indeed encountered compounds that inhibited growth >10%. As the reviewer rightly suggests, this sentence refers only to the compounds that were potential hits from the screen. The results in question are simply confirmatory as we retested biofilm and growth format in lower throughput assays to confirm our findings. Page 14, line 352, the paragraph has been rewritten and now reads: “All five hits did not significantly alter the growth kinetics of the two strains tested in preliminary assays. Planktonic cell growth of EAEC 042 and MND005E in the presence of 0.3125 – 20 μM of hits over a time course of 0 to 8 h was not significantly different from the compound-free control (Figure 2)”. 6. We apologise for writing ‘up to’. The sentence in question should have read at least 30%/ Page 15, line 371-372, the section has now been expanded to describe the potency of individual hits tested with respect to the least concentration that produces biofilm inhibition up to the cut-off mark (30%). The statement now read “Individual analysis of concentration-dependent curves revealed that hits exhibited potent activity, with concentrations as low as 2.5 µM inhibiting biofilm formation by up to 30 % in MMV687800, and much lower (0.3125 µM), resulting in up to 30 % biofilm inhibition in MMV688978 and MMV688990 respectively (Figure 3).” 7. As the reviewer notes, some of our compounds resulted in increased growth. Explanations for this include the possibility that the compound in question serves as a nutrient source (our biofilm assays are done in a high-glucose minimal medium and that organisms that would otherwise be trapped in a biofilm are ‘released’ to the planktonic culture. Experiments on cellular metabolism would probably represent the best way to tease apart these possibilities, however we lack the resources to perform such. In response to the reviewer’s suggestion, we have added a paragraph to the discussion (page 25, line 643-648), pointing out this observation and potential explanations. We have also discussed there that these are factors that need to be explored and dealt with if any of the leads are progressed and that this could be done by combining antibiofilm agents with one another, or with antibacterials. 8. Page 43, details now provided on Table 1 to cover hits for strains 042 and MND005E represented in table. The new title now read “Chemistry of validated 042 and MND005E hits: structures, and molecular descriptors. Data provided by MMV and PubChem” 1. Page 7, line 170-171, dilutions were performed in DMSO, and we understand and agree with the reviewer’s point. To verify possible interference, we re-ran few experiments with dilutions in DMEM. The results for instance with concentration dependent inhibition by three of our hits (MMV687800, MMV688990, and MMV688978, are comparable to those reported when we made dilutions in DMSO. Results are shown as percentage biofilms inhibited by three hits at various concentrations tested (in DMEM / DMSO) in the table below for review purposes.   **Comparison between percentage biofilm inhibition by hits diluted in DMEM and DMSO**     2. The primary paper describing our diarrhoeal study has not yet been published, which is why we cited the conference abstract. The genomes have been submitted to Genbank under bioproject PRJEB8667. This is now indicated in the manuscript on page 6 and 7, line 155-156. Accession numbers for the genomes now posted on ENA and Genbank are listed on Page 45, 3. Line numbers now included in the re-submitted version of manuscript. Page 7, line 178, ‘net’ now changed to neat.   Page 21, line 537, an ‘a’ has now been added before ‘screen’. |
|  |  |
| **REVIEWER 4** |  |
| **Summary and General Comments**  In this manuscript, the authors obtained five compounds that could inhibit the formation of EAEC biofilms but did not inhibit the proliferation of EAEC by screening the Medicines for Malaria Venture Pathogen Box, and discussed their mechanism of action. EAEC AaP was identified as the target of MMV687800, one of the biofilm-inhibiting compounds. This is of positive significance for the development of EAEC biofilm inhibitors to prevent EAEC colonization, thereby preventing and treating EAEC diarrhea. However, there are still some problems in this manuscript that need to be revised or answered.  1. No line number is marked in this paper, which is not conducive to manuscript review;  2. The expression of some content in the abstract is inconsistent with the meaning of the expression in the main text. For example, the meaning of "Hits exhibited at least 10-fold greater antibiofilm activity than nitazoxanide" in the abstract is inconsistent with that of "the Five validated Hits in this"Screen were found active at least 10 times lower than those of nitazoxanide (NTZ) " in the main text. Moreover, according to Figure 1, hits had a good anti-biofilm activity when the concentration was close to 10% of NTZ (Hits 5µM, NTZ48.8µM), but it did not mean that the anti-biofilm activity of hits was at least 10 times that of NTZ. Because the biofilm inhibition rate at the known minimum effective concentration of NTZ is close to 50%, a further 10 times higher biofilm inhibition rate is not possible.  3. The manuscript is not rigorous enough, there are some instructions or labeling errors. For example, in P13, "in Figure 1c" should be corrected to "in Figure 1d"; In Figure 5A, "*" should be marked on SB1 instead of LV1; In Figure 6, there is no data of 3.1.14 strains and SB1 strains in the Figure, so 3.1.14 strains and SB1 strains should not appear in the legend.  4. Why is the OD value in Figure 6 much higher than that in Figure 5? Please check the data carefully and give a reasonable explanation.  5. Why is the effect of biofilm inhibition in Figure 5 expressed by OD value instead of biofilm inhibition rate? Please use biofilm inhibition rate as ordinate to make statistical graph and supplement in the manuscript. The content related to Figure 5 is not clearly expressed in the text and Figure 5. Please review it carefully, conduct statistical analysis again, and make it clear. Statistical P value should be indicated in the figure or text if significant difference is involved in comparison. Please provide relevant original data for statistical experts and reviewers to check again. | We thank the reviewer for this feedback.   1. Line numbering now included in the re-submitted version. 2. We apologise for the incoherence. Page 2, line 66-67, the section in abstract now reads "Hits exhibited potent antibiofilm activity at concentrations at least 10-fold lower than those reported for nitazoxanide, the only known EAEC biofilm inhibitor”, which is expressed in agreement with the body text statement on page 13, line 330-331 (Additionally, the five validated hits in this screen were found active at concentrations at least 10 times lower than those of nitazoxanide (NTZ) as shown in Figure 1d). 3. Page 13, line 331, ‘in Figure 1c’ now corrected to ‘in Figure 1d’, in page 38, Figure 5a, "*" now marked on SB1 instead of LV1, in page 39, Figure 6, information on 3.1.14 and SB1 has now been deleted from the legend. 4. We apologize for this oversight and thank the reviewer for pointing it out. The ODs in Figure 5 were read after diluting crystal violet eluate by factor 2.67. This dilution factor was not corrected in the OD values at the time of plotting Figure 5. The values are higher in Figure 6 as the ODs were already corrected with the dilution factor before plotting. In both graphs, however, percentage inhibitions are very similar for each strain. Page 38, the ODs in Figure 5 have now been corrected with dilution factor and the graphs have been replaced with the corrected versions. 5. Data was expressed as OD values rather rates to allow statistical comparison and display of level of significance. Relevant original data has been attached as supplementary data for review purpose. |
