# Supplementary material for: Antibiofilm agents with therapeutic potential against enteroaggregative Escherichia coli
Source: PLoS Negl Trop Dis. 2022 Oct 6;16(10):e0010809. doi: 10.1371/journal.pntd.0010809 (PMC9578610; doi:10.1371/journal.pntd.0010809)
Supplement: Attachment — Submitted filename: Reviewers response round 2.docx [file pntd.0010809.s006.docx]

| Reviewer’s Comment | Author’s Responses |
| --- | --- |
| Reviewer #3: PNTD-D-21-01605R1 Revision Review: |  |
| 1. In this revised manuscript, the authors have dealt with many of my comments as well as those of the other reviewers, and I thank them for this. The manuscript is much improved as a result. 2. Although the authors have now added line numbers to the tracked changed version of the manuscript, the lines they refer to in their rebuttal table do not appear to correspond to these line numbers (in some cases where I was able to track their change, it was to over 100 lines and multiple pages different from that stated), meaning it is still very difficult to track the changes and comments they refer to in many instances. 3. Moreover, there are still some points which have not yet been adequately addressed.   (i). I asked the authors to replace dynamite plots, because they are well known to be misleading, and no longer considered best practice for open data reporting and transparency. The authors have reformatted only one of their figures, introducing a box plot for Figure 9, and for this I thank them. I am also grateful that the authors now more clearly define what their error bars mean. However, a number of the remaining figures (1d, 4a-c, 5a-d, 6) are still presented as dynamite plots, and the authors provide no justification for fixing one plot but not fixing any of the others. Please change these figures.  (ii). I thank the authors for explaining in their rebuttal why they did not include compound MMV687696 in their concentration dependant assays. I can appreciate that gaining access to some compounds may be challenging (although this does present a challenge if something is proposed as a candidate for treatment). Despite their response stating that this compound could not be procured, and their statement that this is mentioned on page 22 lines 564-565, I regret that I could find no mention of this within the revised manuscript, either at the lines specified or elsewhere. I apologise if I have overlooked something, but it would be good to make this point clear in the manuscript, so others can see it.  (iii). I also asked the authors to revise Table 1, because the columns “Percentage biofilm inhibition” and “Percentage growth inhibition” each contain a single value, and it was not clear what this is (and still is not). The authors have now revised the table heading to state that this is for “042 and MND005E”, but this still does not explain what those single values per cell are – are they the mean, median, value for a specific strain (where tested singly)? Changing the title has not resolved this query – please clarify what the table is showing in those columns, and if necessary expand it into two columns (one for each strain).   (iv). Regarding the DMSO, I am glad that the authors have performed additional validation to show that DMSO does not have a strong effect on biofilm inhibition (although the table in their rebuttal indicates that actually DMSO may have a substantial effect on MMV688990) – shouldn’t this data be presented within the manuscript or as a supplement, rather than merely as a response to reviewers?    (v). Furthermore, although the authors have partially addressed the issue about DMSO concentration, the manuscript text does not fully reflect this – the revised Methods for the inhibition assays still state that “5 μl of 200 μM drug solutions dissolved in neat DMSO…” was used with “195 μl of high glucose Dulbecco’s Modified Eagles Medium”. This is still different from the controls, yet it is still preceded by the statement “In every set up, final concentration of DMSO did not exceed 1%”, which is not correct. Please adjust the text to more accurately reflect what was done. | 1. We thank the reviewer for this feedback. 2. Line numbers have been cross checked here to ensure that they are exact matches in both text and reviewer’s response. 3.   (i). Figures 1d, 5a-d and 6 have now been replaced with dots plots. Figure 4a-c been time course assays which should reveal trend were replaced with line graphs. Median of replicates were plotted their ranges were used as error bars. The raw data for all replicates for these graphs have however been provided alongside data for all graphs.  (ii). We apologize that the sentence been refereed was not completely clear for MMV687696. Page 20, lines 487-490, full sentence now reconstructed as “For three of the compounds (MMV687800, MMV688978 and MMV688990) for which we could get sufficient chemical for further testing (unlike MMV687696, a novel compound from MMV, which was not commercially available), inhibition was largely concentration dependent, again suggesting that specific biofilm factors are targeted”. The concern about treatment candidates is noted. However, it should be acknowledged that hits from screens are typically the foundation for lead compounds that may have different structures and the synthesis of the compounds and/or their derivatives can be commissioned outside resource-limited settings like ours.  (iii). Page 36, Table 1 has now been revised appropriately to include headings that clearly separate hits for each of the two strains tested as well as their biofilm and growth inhibitions by respective hits.  (iv). The table containing data comparing dissolutions of compounds in DMSO and DMEM has now been included as supplementary data. It is captured as “Table S1: Comparative concentration dependent antibiofilm activity of three compounds diluted in DMEM and DMSO”.  (v). The reviewer is correct in observing that the highest concentration of DMSO in any given well would approach 2.5%, and not 1% as was stated in the previous version of the manuscript. To address the error and indicate how much confounding could be contributed from solvent effects, we have edited the section on lines 155-158 to read, “In every set up, up to 5 μL of DMSO was included in each 200 μL well so that the final concentration of DMSO did not exceed 2.5%, which we confirmed in preliminary and follow up assays (Table S1) had a very minor effect on EAEC growth and biofilms. Control wells containing 1% DMSO were therefore included in each experiment. “ |
